# The Vulnerable Conduit: Uncovering Segment-Specific Pathological Susceptibility and Bony Co-dependence of the Vertebral Artery in a South Indian Cohort

**DOI:** 10.7759/cureus.104654

**Published:** 2026-03-04

**Authors:** Prasath Siva, Jayasree Srinivasan, Kalpana Ramachandran, Haripriya M

**Affiliations:** 1 Anatomy, Sri Ramachandra Institute of Higher Education and Research, Chennai, IND

**Keywords:** anatomical variations, computed tomography angiography, foramen transversarium, vertebral artery, vertebral artery hypoplasia

## Abstract

Background

The vertebral artery is a vital contributor to posterior cerebral circulation and demonstrates considerable anatomical variability due to its complex embryological development and close relationship with the cervical vertebrae. These variations have important clinical implications during cervical spine surgery, endovascular procedures, and in the assessment of vertebrobasilar insufficiency. However, comprehensive population-specific data on vertebral artery morphology in South Indians remain limited. The present study aimed to evaluate the anatomical variations, morphometric characteristics, and pathological associations of the vertebral artery using three-dimensional computed tomography angiography (3D-CTA) in a South Indian cohort, with special emphasis on segment-specific vulnerability and its relationship with the foramen transversarium.

Methodology

This retrospective observational study included CTA images of 151 adult patients (100 males and 51 females) obtained between December 2024 and March 2025. Three-dimensional volume-rendered and multiplanar reconstructed images were analyzed to assess vertebral artery origin, level of entry into the foramen transversarium, segment-wise diameter (V1-V4), vertebral artery hypoplasia (≤2 mm), foramen transversarium dimensions, and associated pathological changes. Measurements were independently reviewed by two radiologists. Statistical analysis was performed using SPSS version 19.0 (IBM Corp., Armonk, NA, USA), and a p-value <0.05 was considered statistically significant.

Results

Normal origin of the vertebral artery from the subclavian artery was observed in 95.4% of cases, while anomalous origin was identified in 4.6%. Entry into the foramen transversarium most commonly occurred at the C6 level (96.7%), with high or low entry levels observed in a small minority of cases. No significant association was found between vertebral artery origin and entry level. Segment-wise diameter analysis revealed no significant side-to-side or sex-based differences. The intracranial V4 segment demonstrated the largest mean diameter bilaterally and was the only segment showing significant variation across pathological categories (p = 0.033). Vertebral artery hypoplasia was present in 47.0% of cases, with a right-sided predominance. A significant positive correlation was observed between the diameter of the V4 segment and the corresponding foramen transversarium diameter on both sides (p = 0.0001), whereas no such correlation was found for the extracranial segments.

Conclusions

Although standard vertebral artery anatomy predominates in this South Indian cohort, clinically significant morphometric variations, particularly vertebral artery hypoplasia and intracranial segment vulnerability, are common. The observed correlation between V4 caliber and foramen transversarium dimensions highlights a developmental vascular-osseous interdependence. Although this study carries certain limitations, such as the retrospective nature of the study and the clinically referred cohort undergoing evaluation for cerebrovascular pathology, which may account for the higher observed prevalence of vertebral artery hypoplasia, these findings underscore the importance of detailed preoperative imaging to improve diagnostic accuracy and surgical safety in vertebrobasilar pathologies.

## Introduction

The vertebral artery is a vital component of the human vascular system, providing the primary arterial supply to the brainstem, cerebellum, and posterior cerebral regions. Generally originating as the first and largest branch of the subclavian artery, it undergoes a complex course from the thoracic inlet to the intracranial region. Understanding the morphology of the vertebral artery is essential for neurosurgical precision and diagnostic accuracy in the management of vertebrobasilar pathologies [1-3].

Clinical practice standardizes the vertebral artery into four segments based on its spatial relationship with the cervical spine and cranium [4]. V1 (preforaminal) extends from the subclavian origin to the point of entry into the transverse canal, most commonly at the C6 level [2]. V2 (foraminal) ascends vertically through the foramina transversaria of the upper six cervical vertebrae. V3 (suboccipital) winds medially around the lateral mass of the atlas (C1) within the suboccipital triangle before piercing the dura mater [5]. V4 (intracranial) enters the skull through the foramen magnum and ascends along the medulla to converge with the contralateral vertebral artery at the lower pontine border, forming the basilar artery [1].

During the fourth and fifth weeks of embryonic gestation, the vertebral artery forms through a specialized adaptation of the primitive intersegmental vascularization pattern. The morphogenesis involves three distinct developmental components [4,6]. The proximal portion (V1) arises from the dorsal division of the persistent seventh cervical intersegmental artery [6]. The intermediate portion (V2) develops from a series of vertical postcostal anastomoses that link the first six cervical intersegmental arteries [3,5]. The distal portion (V3 and V4) derives from the proatlantal artery and the spinal branches of the first cervical intersegmental artery [4]. Following the establishment of these longitudinal connections, the proximal segments of the first through sixth intersegmental arteries regress, leaving a continuous arterial trunk. As the vertebrae undergo chondrification and ossification, the artery becomes encased within the foramina transversaria [3,6,7].

Anatomical deviations from the standard course are frequent and clinically significant. Left vertebral artery origin directly from the aortic arch is the most common anomaly, occurring in 2.4%-5.8% of individuals. Right-sided anomalies are rarer (0.7%) and often associated with an aberrant right subclavian artery. Additionally, the artery may enter the transverse canal at high levels (C4 or C5) in up to 20% of cases, increasing the risk of iatrogenic injury during anterior cervical discectomy and fusion (ACDF) [1,6-8]. Vascular symmetry is observed in only 26% of the population. Marked differences in diameter, known as vertebral artery dominance (VAD) or hypoplasia (diameter <2 mm), can alter cerebral hemodynamics. These variations are linked to an increased incidence of posterior circulation infarcts due to “dead spot” thrombosis at the vertebrobasilar junction [1,8].

This study aims to perform a segment-wise morphometric evaluation of the vertebral artery and assess its association with transverse foraminal dimensions in a defined cohort. The study is comprehensive in scope, examining vertebral artery origin, level of entry, segmental diameters, hypoplasia, foramen transversarium dimensions, and pathological associations. This study also involves assessment of correlations between vascular and osseous parameters and evaluation of associated pathological findings.

## Materials and methods

Study design and setting

This retrospective, observational, imaging-based study was conducted at the Department of Anatomy in collaboration with the Department of Radiology, Sri Ramachandra Institute of Higher Education and Research (SRIHER), Chennai, India. The study period extended from December 2024 to March 2025. Ethical approval was obtained from the Institutional Ethics Committee of SRIHER (approval number: CSP-111/25/FEB/17/104). As this was a retrospective study utilizing anonymized imaging data, the requirement for informed consent was waived.

Study population

Computed tomography angiography (CTA) images of 151 adult patients who underwent vertebral artery evaluation during the study period were included. The majority of patients were referred for evaluation of suspected or established cerebrovascular pathology.

Inclusion and exclusion criteria

Patients aged between 18 and 80 years who underwent CTA with adequate visualization of the vertebral arteries were included in the study. Patients with a history of cervical vascular surgery or interventions affecting the vertebral arteries were excluded. Cases with congenital or acquired pathologies unrelated to vertebral artery variation, as well as CTA images with inadequate quality or incomplete visualization of the vertebral artery, were also excluded.

Image acquisition

CTA images were retrieved from the institutional radiology database. Imaging was performed using standardized angiographic protocols on multidetector CT scanners, including the GE Revolution EVO 128-slice CT scanner and the Philips Brilliance 16-slice CT scanner. Three-dimensional volume-rendered images and multiplanar reconstructions were used for detailed anatomical assessment.

Image evaluation and data collection

All CTA images were independently reviewed by two experienced radiologists using a Picture Archiving and Communication System workstation. The radiologists were aware of the clinical indication but were blinded to each other’s observations. Any discrepancies were resolved by mutual consensus.

Each vertebral artery was systematically evaluated for anatomical parameters. The origin of the vertebral artery was classified as normal when arising from the subclavian artery and as a variant when originating directly from the aortic arch or other anomalous sources. The level of entry into the foramen transversarium was recorded as normal when occurring at the C6 vertebra, high entry when above C6, and low entry when below C6.

Segment-wise diameter measurements were performed for both right and left vertebral arteries. The V1 segment, extending from the origin to the entry into the foramen transversarium, was measured at the midpoint between the origin and the transverse foramen entry [9]. The V2 segment, representing the foraminal segment from C6 to C2, was measured between the C3 and C4 vertebral levels, where the artery is relatively straight and uniform [9]. The V3 segment was measured along the vertical portion just before entry into the dural ring [10]. The V4 segment, representing the intracranial portion, was measured using oblique CTA views at a point 11 mm cranial to the entrance of the foramen magnum [11]. All measurements were recorded in centimeters.

Vertebral artery hypoplasia was defined as an arterial diameter of 2 mm or less at any segment, and the laterality of hypoplasia was documented [11]. The foramen transversarium diameter was measured as the maximum anteroposterior distance on axial CT images at the corresponding vertebral level [12]. Pathological associations related to the vertebral artery were also documented, including atherosclerotic changes, vessel narrowing, hypoplasia, dilatation, and altered opacification.

Statistical analysis

Statistical analysis was performed using SPSS Statistics version 19.0 (IBM Corp., Armonk, NY, USA). Categorical variables such as sex, vertebral artery origin, entry level, hypoplasia, and pathological associations were expressed as frequencies and percentages. Continuous variables, including vertebral artery and foramen transversarium diameters, were expressed as mean ± standard deviation. Non-parametric statistical tests were applied due to the data distribution. The Mann-Whitney U test was used for comparison between genders, while the Wilcoxon signed-rank test was used to compare right and left sides. Fisher’s exact test was applied to assess associations between categorical variables, and the Kruskal-Wallis test was used for comparisons across pathological groups. The relationship between vertebral artery diameter and foramen transversarium diameter was evaluated using Spearman’s correlation test. A p-value of less than 0.05 with a confidence interval of 95% was considered statistically significant.

## Results

Study population

A total of 151 CTA studies were analyzed in this study. The cohort consisted of 100 (65.7%) males and 51 (34.3%) females, with a predominance of individuals aged 40-79 years (84.8%).

Vertebral artery origin

**Figure 1:**
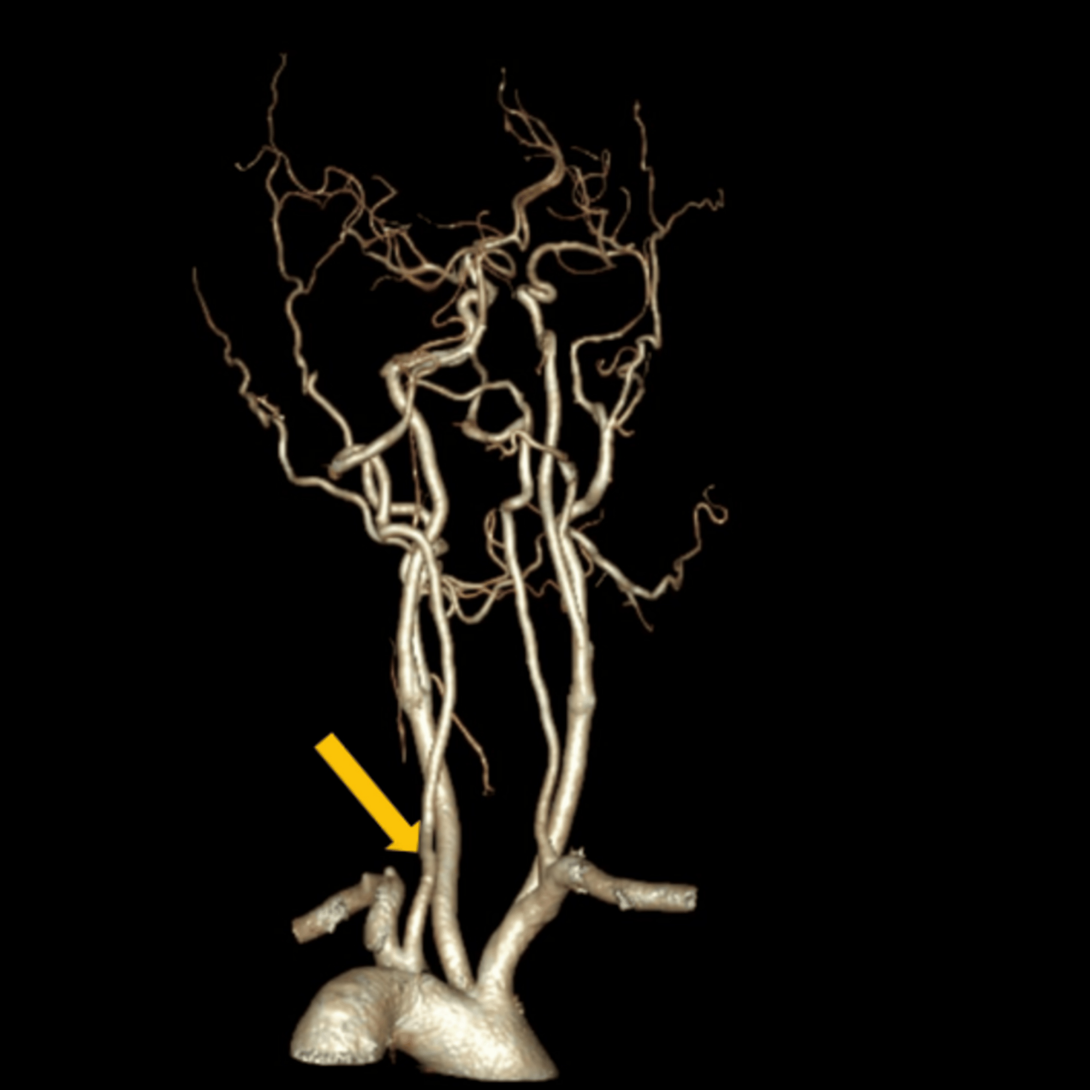
Origin of the vertebral artery from the arch of aorta. The yellow arrow indicates the vertebral artery.

A typical origin of the vertebral artery from the subclavian artery was observed in 144 (95.4%) cases, while seven (4.6%) cases demonstrated an anomalous origin (Figure 1). No statistically significant association was found between the origin of the vertebral artery and gender (p = 1.000).

Level of entry into the foramen transversarium

**Figure 2:**
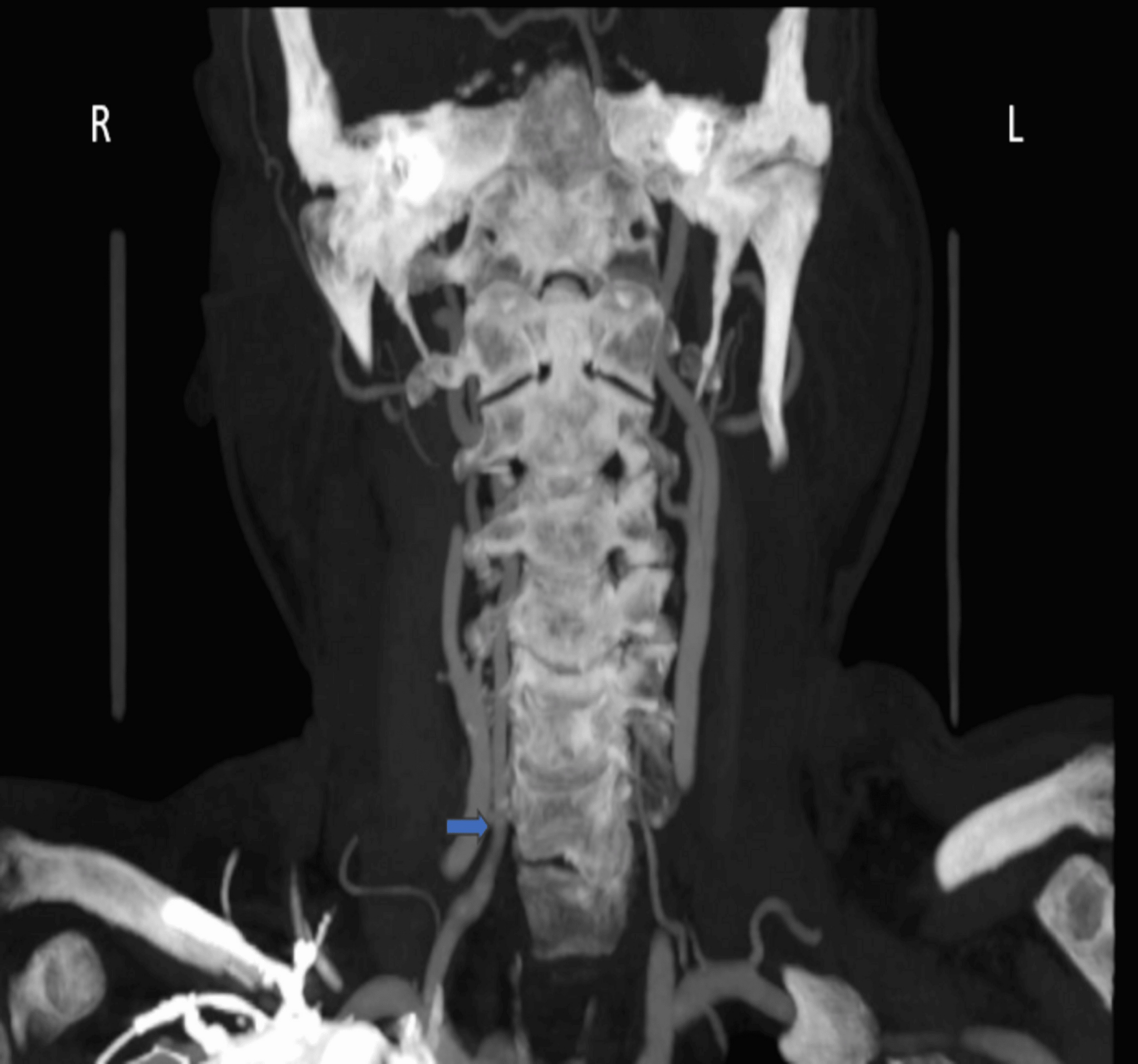
Low entry of the vertebral artery into the foramen transversarium (blue arrow).

The vertebral artery entered the foramen transversarium at the C6 level in 146 (96.7%) cases. High entry (above C6) was noted in two (1.3%) cases, and low entry (below C6) in three (2.0%) cases (Figure 2). There was no significant association between vertebral artery origin and level of entry (p = 1.000).

Vertebral artery diameter

The mean diameters of the vertebral artery segments (V1-V4) showed no statistically significant side-to-side differences (all p > 0.05). Similarly, no significant sex-based differences were observed across all segments (Table 1). The V4 segment demonstrated the largest mean diameter bilaterally compared to other segments.

**Table 1:** Comparison of mean diameter of vertebral artery segments (cm) (Wilcoxon signed-rank test).

Segment	Right (mean ± SD)	Left (mean ± SD)	*Z value*	*P*-value
V1	0.177 ± 0.029	0.173 ± 0.025	-0.909	0.364
V2	0.185 ± 0.034	0.186 ± 0.034	-0.345	0.730
V3	0.142 ± 0.087	0.149 ± 0.011	-0.299	0.765
V4	0.411 ± 0.108	0.410 ± 0.098	-0.376	0.707

Vertebral artery hypoplasia

**Figure 3:**
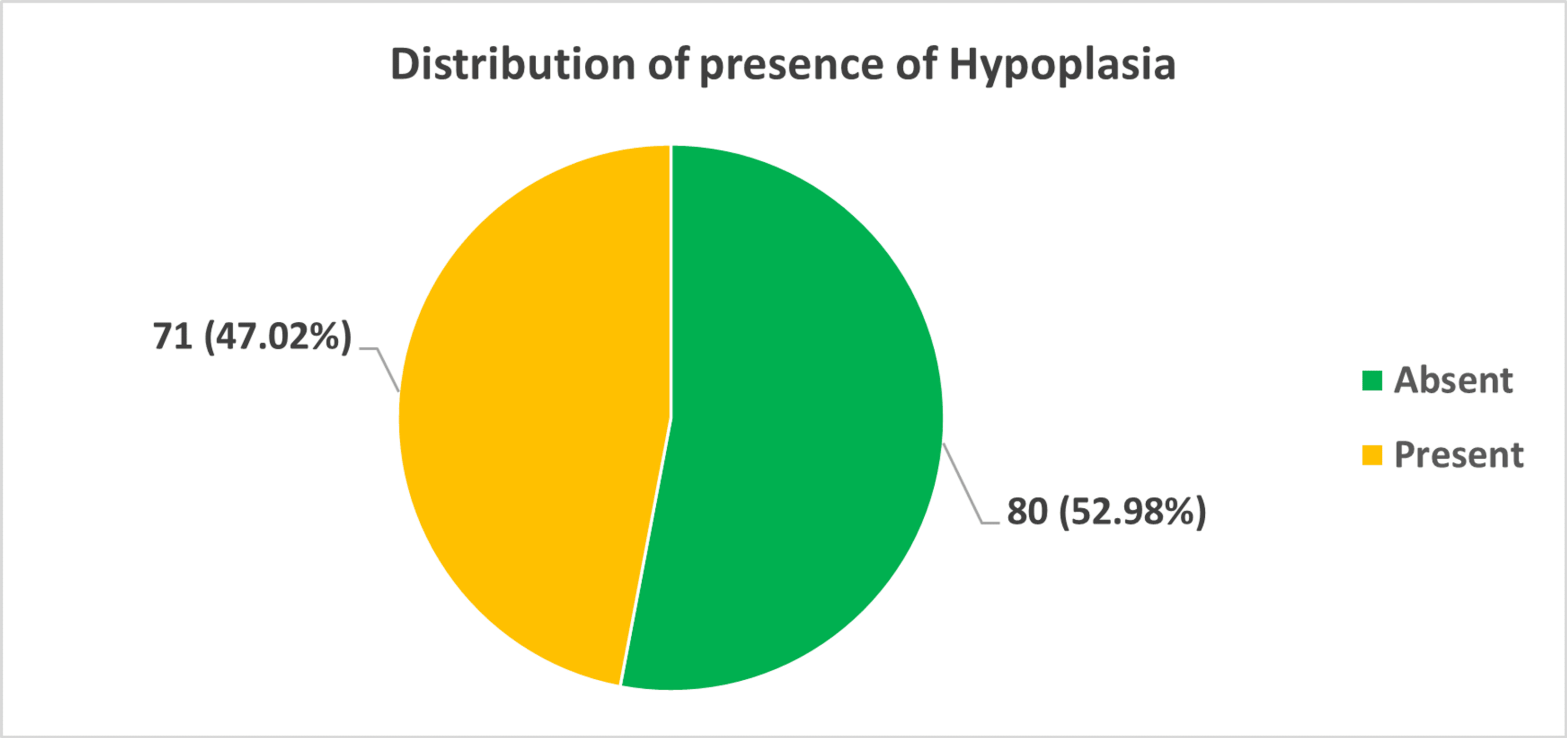
Distribution of vertebral artery hypoplasia.

Vertebral artery hypoplasia (≤2 mm) was identified in 71 (47.0%) cases. Right-sided hypoplasia was more common (27.8%) than left-sided (19.2%) (Figure 3).

Foramen transversarium dimensions

The mean diameter of the foramen transversarium was 0.455 ± 0.088 cm on the right and 0.452 ± 0.079 cm on the left, with no significant side or sex differences (p > 0.05).

Correlation between vertebral artery and foramen diameter

A significant positive correlation was observed between the V4 segment diameter and the corresponding foramen transversarium diameter (right V4: r = 0.512, p = 0.0001; left V4: r = 0.458, p = 0.0001). No significant correlations were identified for V1-V3 segments.

Pathological associations

**Figure 4:**
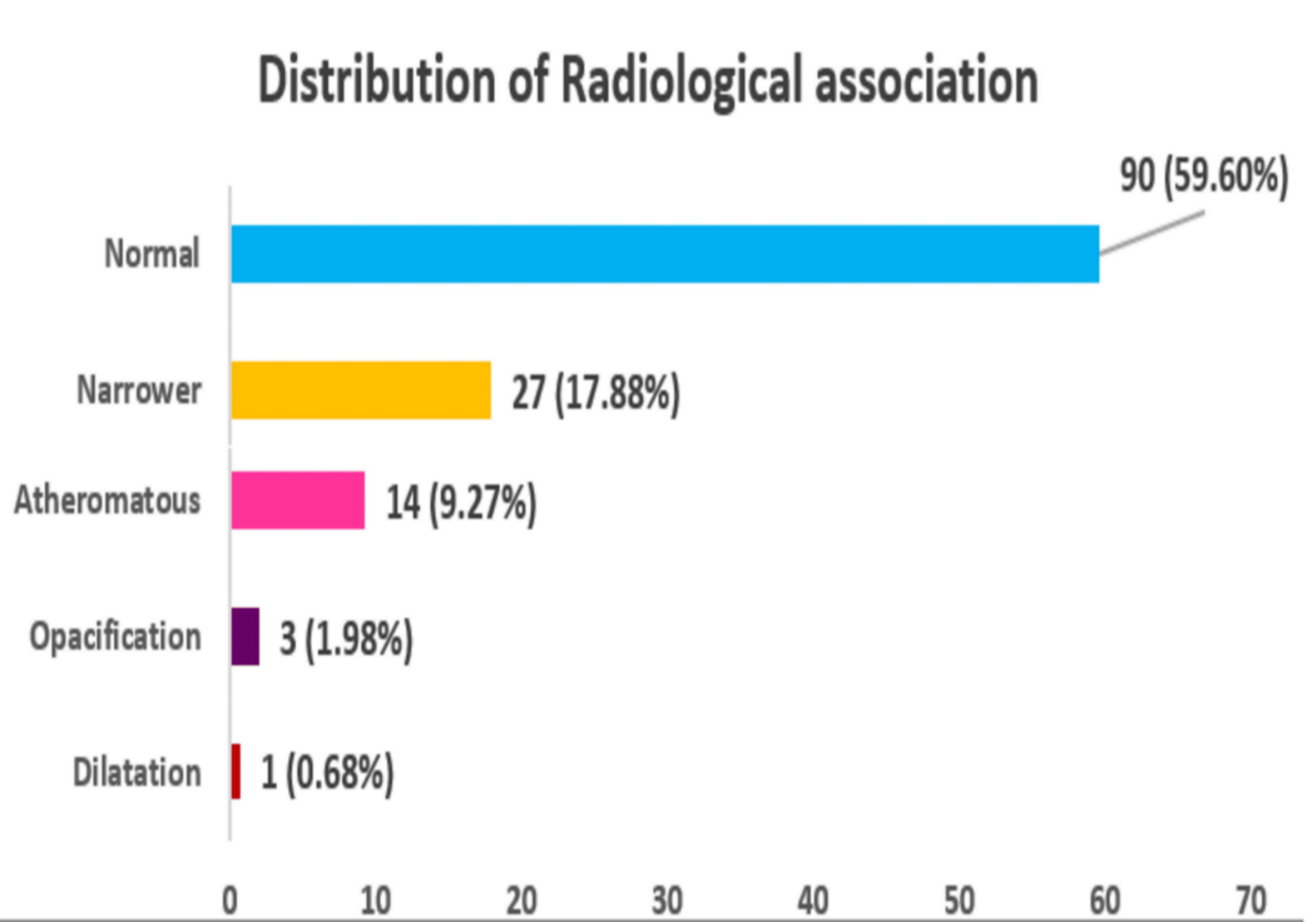
Distribution of radiological-pathological associations.

Most cases were radiologically normal (59.6%). Pathological findings included vessel narrowing (17.9%), hypoplasia (10.6%), atheromatous changes (9.3%), opacification (2.0%), and dilatation (0.7%) (Figure 4). Among vertebral artery segments, only the V4 segment showed a statistically significant difference in diameter across pathological categories (p = 0.033). No significant differences were observed for V1-V3 segments.

Key findings

Normal vertebral artery anatomy predominated in this cohort, with anomalous origin observed in only 4.6% of cases and abnormal entry level in 3.3%. This confirms that major vertebral artery variants remain uncommon within this specific population. The C6 vertebral level served as the most frequent site of entry into the foramen transversarium, accounting for 96.7% of cases. Furthermore, no statistically significant association was identified between the origin of the vertebral artery and its specific entry level.

Segment-wise measurements of the vertebral artery diameters (V1-V4) demonstrated symmetry, with no significant differences observed between the right and left sides or based on gender. However, the study revealed a high prevalence of vertebral artery hypoplasia at 47.0%, showing a right-sided predominance. This high rate likely reflects the nature of the study population, which was enriched with patients referred for cerebrovascular disease evaluation.

A significant positive correlation was identified between the vertebral artery caliber and the foramen transversarium diameter, though this relationship was exclusive to the V4 segment; no such association was observed for the V1-V3 segments. Additionally, pathological changes predominantly affected the intracranial (V4) segment. This segment demonstrated significant diameter variation across various radiological categories, a finding that distinguished it from the more stable extracranial segments.

## Discussion

As the main route for the posterior cerebral circulation, the vertebral artery complex is still one of the human body’s most anatomically varied and clinically important vascular systems. The current study offers a thorough 3D-CTA analysis of the vertebral artery in a clinical cohort from South India, revealing significant rates of morphological variability that differ from traditional anatomical descriptions. The main results of this investigation highlight a high frequency of vertebral artery hypoplasia and a segment-specific pathological vulnerability in the intracranial (V4) section of the vessel, despite a preponderance of conformity to standard origin and entry patterns. Significant new information about the developmental co-dependence of the vertebrobasilar system and its bony encasement is provided by the discovery of a strong positive correlation between the diameter of the V4 segment and the corresponding foramen transversarium dimensions, which is absent in the extracranial segments. These findings have significant ramifications for the diagnostic classification of patients at risk for posterior circulation ischemia, endovascular intervention, and neurosurgical precision.

Comparative analysis of origin and embryological morphogenesis

In this South Indian cohort, the prevalence of anomalous vertebral artery origin was found to be 4.6%, which is in close agreement with the international meta-analytic range of 2.4%-5.8%. The main variation found is in line with international reports; it is the left vertebral artery that emerges straight from the aortic arch between the left common carotid and left subclavian arteries [1,13,14].

From a developmental standpoint, the failure of the proximal segment of the sixth intersegmental artery to regress and the increased absorption of the embryonic tissue of the left subclavian artery into the aortic sac can account for the direct origin of the left vertebral artery from the aortic arch in 4.6% of this cohort. This variation changes the hemodynamic environment of the vessel, making it more than just an anatomical curiosity. Because the vertebral artery receives blood from the high-pressure aortic arch instead of the lower-pressure subclavian system in people with an aortic origin, the vessel wall may be more susceptible to shear stress and linear flow patterns, which could lead to early atherosclerotic changes or dissection [14-16].

The fact that this study found no statistically significant correlation between sex and vertebral artery origin (p = 1.000) supports the idea that these variants are developmental “accidents” that happen in the early weeks of embryogenesis and are mostly unrelated to sex-linked genetic factors. The aortic origin is still a crucial variation that physicians must recognize before performing thoracic or neck surgery, despite its prevalence being consistent across various studies [17].

Interpretation of variations in entry level and surgical vulnerability

The level at which the vertebral artery enters the foramen transversarium is crucial in cervical spine surgery. In this study, 96.7% of vertebral arteries entered at the C6 level, which is on the higher end of the 88% to 95.87% reported in broader literature. The identification of high entry levels (above C6) in 1.3% of cases and low entry levels (below C6) in 2.0% of cases underscores the existence of a vulnerable minority [18].

A high entry level (C4 or C5) implies that the V1 segment of the artery travels a longer distance in the anterior neck without the bony protection of the transverse processes [19]. During ACDF, surgeons rely on the lateral uncinate process as a landmark to avoid the vertebral artery [20]. If the artery enters at C4 or C5, it may course more medially and anteriorly than expected, placing it at extreme risk of iatrogenic injury during uncinate process removal or the lateral expansion of the surgical site [19]. While the overall incidence of vertebral artery injury in ACDF is relatively low (0.09% to 1.96%), the consequences are often catastrophic, including massive hemorrhage, posterior circulation stroke, or death [21]. The clinical relevance of transverse foramen morphology relates primarily to the lateral extent of bony decompression and instrumentation rather than the superficial anterior surgical corridor.

The findings of the present study showed no significant association between vertebral artery origin and entry level (p = 1.000), which is an interesting deviation from some literature that suggests aortic origin is strongly linked to C5 or C4 entry. For example, other studies have reported that up to 80.4% of vertebral arteries originating from the aortic arch enter at the C5 level [13]. The discordance in the present study may be due to the relatively small number of variant cases (n = 7), or it may reflect a population-specific trait of the South Indian demographic, where entry level and origin are more independent than in other groups. Regardless, this independence emphasizes the necessity of preoperative CTA in every case, as a normal subclavian origin does not guarantee a standard C6 entry [22].

One of the more unique findings of this study was the lack of statistically significant differences in diameter between the right and left vertebral arteries across all four segments. This contrasts sharply with the established consensus in clinical anatomy, which posits that symmetry is observed in only approximately 26% to 29.5% of the population, with left-side dominance being the most common variant [23]. Specifically, global literature often reports the left vertebral artery as being larger than the right, a phenomenon that has been documented in both cadaveric and imaging studies [18,24].

The morphometric measurements across all segments of the vertebral artery are consistent on both sides. The observed symmetry in the present study could be a reflection of the South Indian demographic or an artifact of the clinical recruitment pattern. However, the V4 segment (intracranial) emerged as the largest segment bilaterally (0.411 ± 0.108 cm on the right and 0.410 ± 0.098 cm on the left), which deviates from some reports where the V1 or V3 segments are noted to be larger. In many European and American studies, the vertebral artery caliber is reported to decrease significantly after entering the intracranial cavity [22]. The fact that the V4 segment was the largest in this cohort, as well as the most pathologically affected, suggests a unique structural profile in South Indians that warrants further investigation.

This symmetry suggests that in the South Indian population, the developmental pressures leading to asymmetrical dominance may be less pronounced, or that the clinical population recruited for this study had a specific bias toward vascular pathologies that occur more frequently in symmetrical or balanced systems. Nevertheless, the clinician must be aware that while symmetry is common in this cohort, individual variations such as vertebral artery hypoplasia are still prevalent.

Pathophysiological implications of vertebral artery hypoplasia

A pivotal discovery in this study is the remarkably high prevalence of vertebral artery hypoplasia (47.0%), defined as an arterial diameter of less than 2 mm. This is significantly higher than the 1.9% to 26.5% reported for the general population in the existing literature [24]. The prevalence of 47.0% is likely an overestimation of the general South Indian population due to the study’s focus on a clinical cohort referred for cerebrovascular evaluation (84.8% aged 40-79 years), as vertebral artery hypoplasia is a well-known predisposing factor for posterior circulation ischemia, acting through multiple physiological mechanisms.

The right-sided predominance of vertebral artery hypoplasia observed in this study (27.8% right vs. 19.2% on the left) aligns with international data. This asymmetry is clinically relevant because hypoplastic vessels are associated with impaired regional blood flow and elevated pulsatility indices in the basilar artery [25]. Chronic hypoperfusion in the territory supplied by a hypoplastic vertebral artery can lead to localized “dead spots” at the vertebrobasilar junction, where the absence of flow mixing creates an environment conducive to thrombus formation and accelerated atherosclerosis.

The presence of vertebral artery hypoplasia has also been linked to poor prognostic outcomes in acute stroke patients, with some studies showing that left vertebral artery hypoplasia specifically correlates with higher rates of basilar artery occlusion and a lower probability of achieving functional independence after three months. Given the prevalence of chronic conditions such as diabetes and hypertension in the South Indian population, the combination of vertebral artery hypoplasia and pre-existing vascular risk factors may have a synergistic effect that significantly raises the risk of posterior circulation stroke [26].

Theoretical explanation of the V4-foramen transversarium correlation

A major theoretical contribution of this study is the identification of a significant positive correlation between the diameter of the V4 segment and the corresponding foramen transversarium (r = 0.512 on the right and r = 0.458 on the left), while no such relationship was found for the V1, V2, or V3 segments. This finding is somewhat paradoxical, as the V1 segment is extraosseous and the V2/V3 segments are the ones actually encased within the transverse foramina of the cervical vertebrae.

Existing literature on this topic is conflicting. While some paleontological and anatomical studies suggest that the size of the foramen transversarium can serve as a reliable proxy for vertebral artery size across the entire cervical column (C1-C6) [27]. Other researchers have found that this correlation is weakest at C1 due to its unique biophysical role in neck rotation [28]. The strong correlation identified specifically for the intracranial V4 segment in this study suggests that the developmental influences of the intracranial vessel exert a “downstream” effect on the bony formation of the upper cervical spine, or that the morphometry of the transition zone at the foramen magnum is more closely linked to the terminal caliber of the vessel than the intermediate transforaminal segments [27]. A common developmental mechanism may influence both vascular caliber and transverse foraminal size. Our data do not refute osseous-vascular coupling, but instead suggest variability in its adult morphological expression. Without longitudinal developmental or embryological data, causative interpretations remain speculative.

One plausible mechanism for this correlation is the timing of vertebral ossification relative to vascular growth. During the developmental stage, the vertebral artery is thought to be a prerequisite for the correct formation of the foramen transversarium. In cases of congenital hypoplasia, the lack of mechanical pressure or signaling from the vessel may result in a commensurately small foramen [28]. The lack of correlation in the V2 segment (the foraminal part) in this study might suggest that the extracranial foramen is “oversized” to accommodate movements of the cervical spine, effectively decoupling the immediate relationship between arterial caliber and bony diameter once a certain protective threshold is reached [29].

From a diagnostic perspective, these findings imply that a unilaterally small foramen transversarium on imaging should raise high clinical suspicion for intracranial vertebral artery hypoplasia, whereas a normal-sized foramen transversarium containing a small-caliber vessel is more likely to represent acquired pathology such as atherosclerosis or dissection.

Intracranial atherosclerosis: racial-ethnic context of V4 pathology

The preferential involvement of the V4 segment in pathological changes (9.3% atheromatous changes, 17.9% vessel narrowing) in this South Indian cohort provides strong evidence for the "isolated intracranial atherosclerosis" pattern commonly observed in Asian, African, and Hispanic populations. In contrast to Western populations, where the origin of the internal carotid artery and the V1 segment (subclavian origin) are the primary sites for severe atherosclerosis, Asian populations tend to develop lesions more distally, particularly in the middle cerebral artery and the intracranial vertebral and basilar arteries [30].

The increased susceptibility of the V4 segment may be explained by its structural features. Compared to extracranial arteries, intracranial arteries have a thinner media, fewer adventitia, and no external elastic lamina. When hemodynamic stressors or inflammatory triggers challenge these vessels, their thicker and denser internal elastic lamina may make them more vulnerable to focal narrowing and dissections [31].

Moreover, distinct patterns of wall shear stress are produced by hemodynamic factors at the flow confluence, where the two vertebral arteries converge to form the basilar artery [32]. In patients with vertebral artery hypoplasia or VAD, the disturbed flow at this junction can accelerate plaque development, explaining why the V4 segment in this study showed the most significant diameter variation across pathological categories (p = 0.033).

Study strengths and limitations

This study’s main strength is that it provides a segmental, high-resolution analysis of the vertebral artery in all four of its components using cutting-edge 3D-CTA technology. The study provides a comprehensive understanding of the vascular-skeletal interface by incorporating both foramen transversarium dimensions and arterial morphometry. A high degree of diagnostic accuracy and inter-observer reliability is ensured by using two independent radiologists and resolving disagreements by consensus.

However, to contextualize these findings, a number of limitations must be addressed. First, the cohort is naturally biased toward symptomatic patients with suspected cerebrovascular disease due to the study’s retrospective design and tertiary referral center setting. This likely explains the high prevalence of hypoplasia (47.0%) and may not reflect the incidence in the general asymptomatic South Indian population. Second, the sample size of 151 patients, while robust, may not be sufficient to capture extremely rare anomalies such as bilateral aortic arch origin or right-sided origin from the common carotid artery, which are estimated to occur in fewer than 0.1% to 0.7% of the population. Yet another limitation is that the branching pattern of the vertebral artery has not been studied in detail in the present study. Furthermore, as a cross-sectional study, it cannot establish a causal timeline between the presence of anatomical variants such as vertebral artery hypoplasia and the development of atherosclerotic plaques. Future longitudinal research is required to determine whether a 2 mm diameter represents a physiological threshold for accelerated vascular aging. Finally, while the study measured the foramen transversarium, it did not account for the presence of accessory or double foramina, which have been reported in up to 19% to 23% of Indian vertebrae and may further complicate the course of the vertebral artery. The authors also acknowledge the measurement bias that could have arisen due to the difference in CTA resolution.

Future directions

The results reported here provide a number of directions for further research. To create definitive morphometric nomograms for the vertebral artery in healthy adults, prospective, community-based studies are needed in the Indian population. It will become clearer how vertebral artery hypoplasia functions as an independent versus symptomatic clinical cohort when these results are compared, contributing to the risk of stroke. Furthermore, incorporating computational fluid dynamics modeling will help gain a better understanding of how variations such as high entry levels or aortic origin change wall shear stress and encourage “dead spot” thrombosis at the vertebrobasilar junction. Investigating genetic markers linked to isolated intracranial atherosclerosis in South Asian populations may help identify the molecular underpinnings of the pathological sensitivity of the V4 segment in comparison to Western populations. Lastly, the connection between the V4 segment and the dimensions of the foramen transversarium found in this study calls for additional research into the mechanobiology of vertebral development, which may have implications for both surgical procedures and our comprehension of human evolution.

## Conclusions

This thorough 3D-CTA assessment of the vertebral artery in a South Indian population shows that, although the standard anatomical model for origin and entry is widely used, notable morphometric variations are frequent and have a clinical impact. This population’s distinct neurovascular profile is highlighted by the high incidence of vertebral artery hypoplasia and the intracranial V4 segment’s segment-specific susceptibility to pathological narrowing. A new potential marker for developmental vascular integrity is provided by the strong correlation found in the upper cervical spine between the artery’s terminal caliber and the surrounding bony structure. These findings emphasize how crucial customized preoperative mapping is for today’s physicians. When managing complex vertebrobasilar pathologies, surgeons and radiologists can only guarantee the highest standards of patient safety and diagnostic accuracy by having a thorough understanding of these anatomical nuances.
